# Supplementary material for: Myasthenia Gravis and Thymectomy at a Tertiary-Care Surgical Centre: A 20-Year Retrospective Review
Source: Curr Oncol. 2025 Nov 27;32(12):662. doi: 10.3390/curroncol32120662 (PMC12731372; doi:10.3390/curroncol32120662)
Supplement: Supplementary file 1 [file curroncol-32-00662-s001.zip › curroncol-3904331-supplementary.pdf]

SUPPLEMENTARY TABLES:

Table S1. Distribution of thymic epithelial tumors (TETs) by WHO classification and myasthenia gravis (MG)

Tumor type	WHO subtype	MG present (n)	MG absent (n)	Total (n)
Thymoma	A	7	18	25
	AB	11	66	77
	B1	26	28	54
	B2	16	17	33
	B3	17	24	41
	Missing	4	2	6
Subtotal (Thymoma)		81	155	236
Thymic Carcinoma		2	9	11
Total TETs		83	164	247

Legend: Data are presented as counts. WHO histologic classification applies to thymoma cases only. In cases where two histologic types were reported for the same tumor, the higher-grade subtype was recorded.

Table S2. Distribution of thymic epithelial tumors (TETs) by Masaoka-Koga Stage classification and myasthenia gravis (MG)

Masaoka-Koga	MG present n = 81	MG absent n = 155	Total n = 236
I (%)	28 (34.5)	66 (42.6)	94 (39.8)
IIA (%)	39 (48.1)	65 (42)	104 (44.1)
IIB (%)	6 (7.4)	13 (8.4)	19 (8)
III (%)	6 (7.4)	7 (4.5)	13 (5.5)
IVA (%)	2 (2.4)	2 (1.2)	4 (1.7)
IVB (%)	0	0	0
Missing (%)	0	2 (1.2)	2 (0.8)

Legend: Data are presented as counts (percentages).

Table S3. Comparison of complete myasthenia gravis (MG) remission 6 months post-thymectomy between thymomatous and non-thymomatous MG

MG Complete Remission (6 months)	Non-thymomatous MG (n=76)	Thymomatous MG (n=65)	Total (n=141)	χ^2 (df=1)	*p*-value
No remission (%)	25 (32.9)	21 (32.3)	46 (32.6)	0.005	0.941
Remission (%)	51 (67.1)	44 (67.7)	95 (67.4)		

Legend: Data are presented as counts (percentages). Pearson's Chi-square test was used to compare remission rates between groups.

Table S4: Intraoperative and postoperative complication rates by surgical approach (open vs. minimally invasive) and length of stay.

**Variable**	**Open Surgery N= 288**	**Minimally Invasive N = 132**	**Total N= 420**	***p*-value**
**Intra-operative complications (%)**				
Yes	33 (11.5)	2 (1.5)	35 (8.3)	<0.001
No	252 (87.5)	130 (98.5)	382 (91)	
**Post-operative complications (%)**				
Yes	129 (44.8)	28 (21.2)	157 (37.3)	<0.001
No	158 (54.9)	104 (78.8)	262 (62.4)	
**Length of stay (days, median ± IQR)**	6 (5-8)	3 (2-4)	5 (3-7)	<0.001

Data are presented as counts (percentages) unless otherwise specified.
Three patients undergoing open surgery were missing intraoperative complication data, and one patient was missing postoperative complication data.

Table S5: Distribution of myasthenia gravis (MG) antibody subtypes in thymomatous and non-thymomatous patients

Antibody status	Thymomatous MG	Non-thymomatous MG	Total
AChR positive – n (%)	52 (36.4)	54 (37.7)	106 (74.1)
Musk positive – n (%)	0 (0)	1 (0.7)	1 (0.7)
LRP4 positive – n (%)	0 (0)	0 (0)	0 (0)
Seronegative – n (%)	15 (10.4)	21 (14.7)	36 (25.2)
Total Valid Cases – n(%)	67 (46.9)	76 (53.1)	143 (100)

Legend: Percentages are based on patients with Myasthenia Gravis with available serological results (valid cases, N=143).
